# Structural, Optical and Electrical Properties of HfO_2_ Thin Films Deposited at Low-Temperature Using Plasma-Enhanced Atomic Layer Deposition

**DOI:** 10.3390/ma13092008

**Published:** 2020-04-25

**Authors:** Kyoung-Mun Kim, Jin Sub Jang, Soon-Gil Yoon, Ju-Young Yun, Nak-Kwan Chung

**Affiliations:** 1 Department of Materials Science and Engineering, Chungnam National University, Daejeon 34134, Korea; kkm1215@kriss.re.kr (K.-M.K.); sgyoon@cnu.ac.kr (S.-G.Y.); 2 Department Materials and Energy Measurement Center, Korea Research Institute of Standards and Science (KRISS), Daejeon 34113, Korea; jjs77715@kriss.re.kr (J.S.J.); jyun@kriss.re.kr (J.-Y.Y.)

**Keywords:** HfO_2_ thin film, low temperature, plasma-enhanced atomic layer deposition, electrical properties

## Abstract

HfO_2_ was deposited at 80–250 °C by plasma-enhanced atomic layer deposition (PEALD), and properties were compared with those obtained by using thermal atomic layer deposition (thermal ALD). The ALD window, i.e., the region where the growth per cycle (GPC) is constant, shifted from high temperatures (150–200 °C) to lower temperatures (80–150 °C) in PEALD. HfO_2_ deposited at 80 °C by PEALD showed higher density (8.1 g/cm^3^) than those deposited by thermal ALD (5.3 g/cm^3^) and a smooth surface (RMS Roughness: 0.2 nm). HfO_2_ deposited at a low temperature by PEALD showed decreased contaminants compared to thermal ALD deposited HfO_2_. Values of refractive indices and optical band gap of HfO_2_ deposited at 80 °C by PEALD (1.9, 5.6 eV) were higher than those obtained by using thermal ALD (1.7, 5.1 eV). Transparency of HfO_2_ deposited at 80 °C by PEALD on polyethylene terephthalate (PET) was high (> 84%). PET deposited above 80 °C was unable to withstand heat and showed deformation. HfO_2_ deposited at 80 °C by PEALD showed decreased leakage current from 1.4 × 10^−2^ to 2.5 × 10^−5^ A/cm^2^ and increased capacitance of approximately 21% compared to HfO_2_ using thermal ALD. Consequently, HfO_2_ deposited at a low temperature by PEALD showed improved properties compared to HfO_2_ deposited by thermal ALD.

## 1. Introduction

The semiconductor industry has developed rapidly, and electronic devices have been scaled down. However, scaled-down devices can show many problems, such as direct tunneling, high gate leakage current and poor reliability [1,2]. Therefore, HfO_2_ has been studied to replace conventional SiO_2_ as a high-κ material because of its advantages, such as high density, good ductility and corrosion resistance, as well as its high-k [3,4]. HfO_2_ has mainly been deposited by thermal atomic layer deposition (thermal ALD) because this method produces thin films that are pinhole-free, high density and have low contaminants levels (Carbon, Nitrogen); this process also allows excellent thickness control [5,6,7,8].

Recently, as the importance of wearable devices has increased, low-temperature deposition of HfO_2_ thin films has been required [9,10]. However, HfO_2_ thin films normally must be deposited at around 200 °C because the metal–organic precursors used as sources during the ALD process fully decompose at high temperatures [11]. Many methods have been studied to lower the deposition temperature of HfO_2_ in ALD [12,13,14,15,16]. However, in those studies, HfO_2_ thin films deposited at low temperatures had problems, such as a high level of carbon impurities (5.15%–8.9% carbon impurity in HfO_2_ thin films deposited at 150 °C) or low density (3.7 g/cm^3^ when deposited at 30 °C, 4.1 g/cm^3^ at 50 °C, 4.8 g/cm^3^ at 80 °C and 5.3 g/cm^3^ at 100 °C); these problems cause high leakage current and poor reliability in electronic devices [12,13,14].

Using plasma to produce oxygen radicals with high reactivity can solve these problems, and plasma has been used in other low-temperature deposition processes [17]. Consequently, plasma-enhanced atomic layer deposition (PEALD) can be used to decompose a source at a lower temperature by making atomic oxygen radicals using O_2_ gas as a reactant; this is in contrast to the conventional thermal ALD process, which uses O_3_ as a reactant. In the previous studies, it was found that the electrochemical oxidation potential, a measure of the sensitivity of the oxidation reaction, of atomic oxygen radicals (2.42 V) is higher than that of O_3_ (2.08 V) [18,19,20]. In the ALD process, electrochemical oxidation potential of the reactants indicates the ligand-decomposing power [21,22,23,24]. Higher oxidation potential of reactants enables the low-temperature processes because less thermal energy is required for source decomposition [16].

In this study, HfO_2_ thin films were deposited by PEALD at 80 °C, and their variable properties, such as film structures, surface morphology and surface components, were compared with thin films deposited by using thermal ALD and PEALD at various temperatures (80, 150 and 250 °C). Moreover, values of densities, refractive index, optical bandgap determined by Tauc plot and transmittance of HfO_2_ deposited at 80 °C by thermal ALD and PEALD were compared. In our study, the HfO_2_ deposited at a low temperature (80 °C) by PEALD showed a low carbon ratio (3.5%) and high film density (8.1 g/cm^3^). Finally, electrical characteristics, such as capacitance–voltage (C–V) curve, current–voltage (I–V) and fixed-charge density (Q_f_) of HfO_2_ deposited at 80, 150 and 250 °C were analyzed, using an MOS capacitor. The HfO_2_ thin films deposited at a low temperature (80 °C), using PEALD, showed improved structural, chemical, optical and electrical properties, without any degradation.

## 2. Materials and Methods

Using an automated ALD system (iCV d300, ISAC Research,Daejeon, Korea), HfO_2_ thin films were fabricated on doped (ρ ~ 15 Ω·cm) p-type Si (100) wafers. Substrates were cleaned for 10 min with acetone, 10 min with ethanol and 10 min with IPA in an ultrasonic generator; they were immediately dried by blowing argon over the sample. The substrates were loaded at different temperatures, in a range of 80–250 °C. The main pump was an MVP-90 (WOOSUNG VACUUM PUMP, Jeju, Korea), and the base pressure was 10 mtorr. An ISP-90 (ANEST IWATA Corporation, Yokohama, Japan) was used as a by-pass pump for constant flow. In this experiment, direct plasma was used; the plasma power was fixed at 150 W, using a 13.56 MHz RF power supply (REX2-3K, RF Power Tech, Anyang, Korea). Tetrakis(ethylmethylamino) hafnium (TEMAH-99.999% purity from UP Chemical, Pyungtaek, Korea) was used as a precursor. High-purity O_3_ and O_2_ were used as oxidants. O_3_ was produced from O_2_ by an ozone generator (LAB-II, Ozonetech, Daejeon, Korea). Ar gas, used as a carrier gas and purge gas, also had a purity of 99.999%. TEMAH precursor canister was maintained at 75 °C. The precursor flow line and the chamber were also maintained at 80 °C, to prevent condensation and clogging.

The thickness of the HfO_2_ thin films was measured by using a Reflectometer (ST2000, K-MAC, Daejeon, Korea) and Spectroscopic Ellipsometry (SE, M2000D, J.A. WOOLLAM CO, Lincoln, NE, USA). In addition, the film structures and density in HfO_2_ were examined by Grazing Incidence X-ray diffraction and X-ray reflectivity, respectively (GIXRD, MXD10, Rigaku, Tokyo, Japan, Cu Kα radiation). The root mean square (RMS) roughness values of the HfO_2_ films (50 nm) were obtained by Atomic Force Microscope (AFM, XE7, Park Systems Suwon, Korea) images and scanned at 2 µm × 2 µm size. The chemical bonding states and components were examined by using X-ray photoelectron spectroscopy (XPS, K-Alpha+, Thermo Fisher Scientific Waltham, MA, USA) To remove carbon- and nitrogen-contaminant layers from air, approximately 7 to 10 nm of the HfO_2_ films was removed via Ar etching, at 1 keV, for 30 s [25,26]. Refractive index and absorption coefficient of HfO_2_ (50 nm) were extracted from the Ellipsometry (SE, M2000D, J.A. WOOLLAM CO, Lincoln, NE, USA) data. The transmittance of HfO_2_ (50 nm) at 550 nm on the PET substrate (ST510, DuPont Teijin Films, Wilmington, DE, USA) was measured in a range from 190 to 1100 nm, which was measured in the normal incidence of light by UV-vis spectroscopy (HP 8453, Agilent, Santa Clara, CA, USA). To measure the electrical properties (I–V and C–V), MOS capacitors were fabricated. Cu/Ti top electrodes were deposited on HfO_2_/p-Si, using an E-beam evaporator (KVET—C500200, Korea Vacuum, Gimpo, Korea). Cu/Ti circular electrodes were patterned, using a shadow mask. Electrical properties, as indicated by the I–V and C–V curves, were measured by using a Manual Probe Station (SUMMIT 11862B, Cascade, Beaverton, OR, USA). The C–V curve was obtained at 1 MHz in the range of −7 to +7 V, and the I–V curve was obtained from −2 to 2 V.

## 3. Results and Discussion

**Figure 1:**
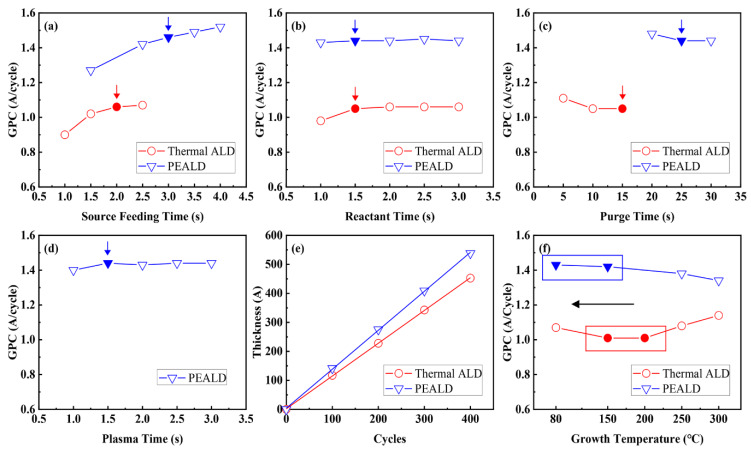
(**a**–**d**) GPC values of HfO_2_ deposited at 80 °C by thermal ALD and PEALD as functions of precursor exposure time, reactant exposure time, purge time and plasma exposure time. (**e**) Thickness values as a function of ALD cycles with thermal ALD and PEALD. (**f**) ALD windows as functions of deposition temperature with thermal ALD (150–200 °C) and PEALD (80–150 °C).

To show the experimental conditions of HfO_2_ thin films deposited at 80 °C, Figure 1a–d provides growth per cycle (GPC) curves for each step time; these were measured by using a reflectometer, because the process is easier and simpler than ellipsometry. 

GPC curves, which changed according to the feeding and purge times, were clearly saturated at the same time with sufficient feeding and purge times. The experimental periods were determined according to these saturation times, as indicated by the arrows in Figure 1a–d. The thermal ALD cycle for HfO_2_ deposition consisted of 2 s source feeding, 15 s Ar purging, 1.5 s O_3_ reactant feeding and 15 s Ar purging. Additionally, the PEALD cycle for HfO_2_ deposition consisted of 3 s source feeding, 25 s Ar purging, 1.5 s O_2_ reactant feeding, a 1.5 s O_2_ plasma-on state and 25 s Ar purging. Since direct plasma was used in the experiment, O_2_ plasma was used for a relatively short time compared with remote plasma. Figure 1e shows the thickness increase with the deposition cycle; resulting values were obtained by ellipsometry, to measure the thicknesses of the thin films, because the reflectometer has difficulty accurately measuring thicknesses under 100 nm. The HfO_2_ thickness increased linearly as the cycle increased, without a growth delay problem; GPC values were similar to those obtained from using the reflectometer.

Figure 1f shows the GPC of HfO_2_ thin film according to the deposition temperatures of the thermal ALD and PEALD processes. The temperature section in which GPC shows constant temperature is called the ALD window and is a problem-free deposition region. The region between 150 and 200 °C is the ALD window in thermal ALD. In PEALD, the ALD window shifted to lower temperatures (80–150 °C) from high temperatures (150–200 °C) because of the high reactivity of O_2_ plasma; this allowed more stable low-temperature deposition. When thin films were deposited at 80 °C, using thermal ALD, GPC increased and exhibited condensation because of the insufficient thermal energy. Above 250 °C in thermal ALD, because of source decomposition due to high thermal energy, the GPC increased as the temperature increased. Conversely, in PEALD, GPC decreased, and desorption occurred in a manner different from that in thermal ALD [27]. The reason for this is that, as the temperature rose, increased ion energy of the plasma promoted etching of the HfO_2_ thin film and caused desorption [28]. 

**Figure 2:**
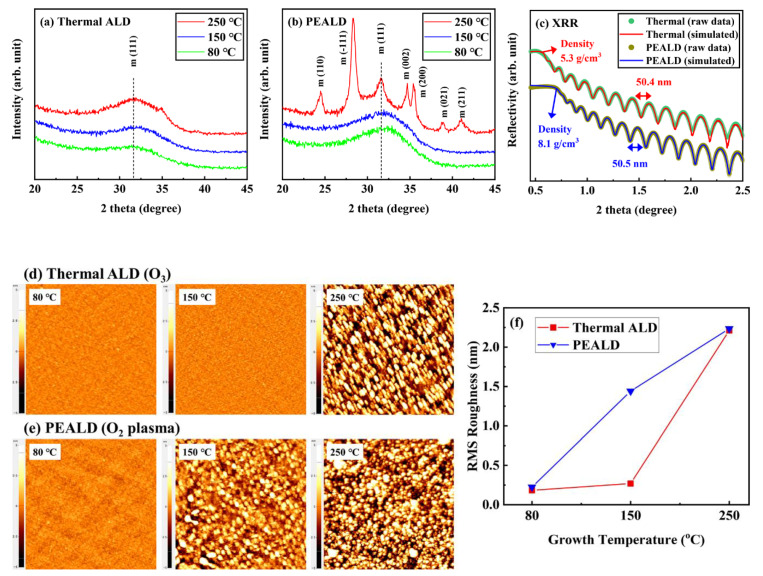
(**a**,**b**) XRD pattern in HfO_2_ thin films (50 nm) deposited at 80–250 °C by thermal ALD and PEALD, measured by GIXRD. (**c**) Density of HfO_2_ thin films (50 nm) formed at 80 °C by thermal ALD and PEALD, measured by XRR. (**d**,**e**) AFM topography images of HfO_2_ thin films (50 nm) deposited by thermal ALD and PEALD. (**f**) Root mean square (RMS) roughness of HfO_2_ by growth temperatures (80–250 °C).

Figure 2a,b shows the XRD pattern of HfO_2_ when deposited by thermal ALD and PEALD [29]. 

XRD patterns of HfO_2_ deposited at 80–250 °C by thermal ALD showed a broad peak at 2θ = 32°, indicating a dominantly amorphous structure HfO_2_ thin film [30]. Moreover, HfO_2_ deposited at 80–150 °C by PEALD also had an amorphous structure, but HfO_2_ deposited at 250 °C by PEALD contained a polycrystalline structure. This means that the crystallization of HfO_2_ thin film deposited by PEALD started at a lower temperature than that of HfO_2_ deposited by thermal ALD [31]. Figure 2c shows the thickness and density of HfO_2_ deposited at 80 °C, obtained from a period and critical angle of reflectivity oscillation pattern, as measured by XRR. Thickness was measured and found to be approximately 50 nm for both thermal ALD and PEALD samples, and density increased in PEALD from 5.3 to 8.1 g/cm^3^. This means that the HfO_2_ thin film deposited by PEALD at a low temperature was denser than that deposited to the same thickness by thermal ALD. Figure 2d–f provides root mean square (RMS) roughness and morphology images of HfO_2_ deposited according to temperature (80–250 °C) in thermal ALD and PEALD. In the PEALD samples, there was no difference of roughness compared to the thermal ALD samples, and the HfO_2_ thin film was still flat at 80 °C (0.2 nm). Additionally, no large particles were seen when HfO_2_ was deposited at a low temperature. As the temperature rose, the roughness of the thin film rapidly increased due to the formation of crystallite [32].

**Figure 3:**
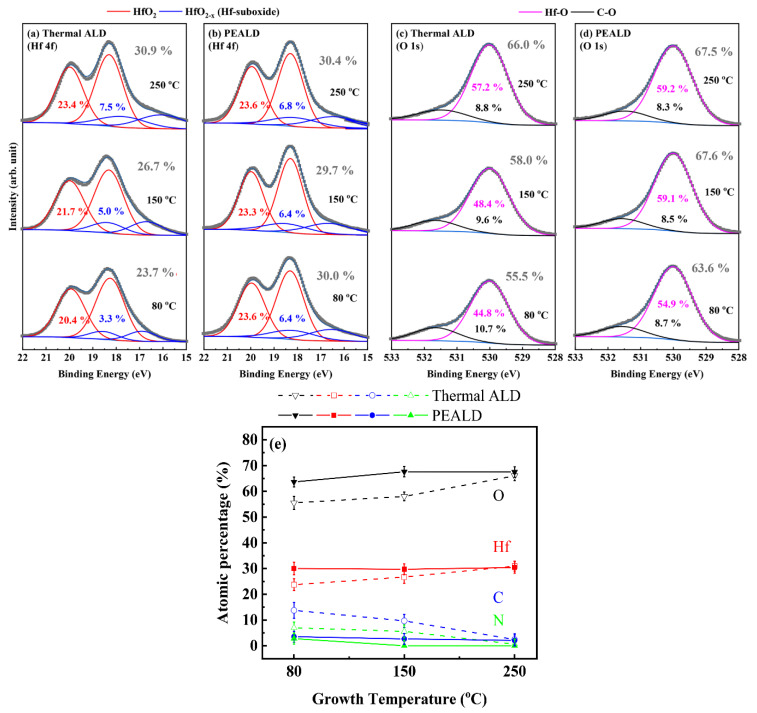
(**a**,**b**) Hf 4f and (**c**,**d**) O 1s spectra of HfO_2_ thin films (50 nm) on Si substrate fabricated by thermal ALD and PEALD. Gray dotted lines and blue solid lines are sum of the spectra before fitting and sum of the deconvoluted peaks after fitting, respectively. (**e**) Surface component percentages of O, Hf, C and N in HfO_2_ thin films (50 nm). The error bars represent the standard deviations.

Figure 3a,b shows XPS results for Hf 4f formed by thermal ALD and PEALD, respectively. 

The deconvoluted Hf 4f spectra show the doublet of peaks at binding energy of 18.31 and 19.99 eV, which is associated with HfO_2_ [33]. Moreover, at binding energy lower than those of the 4f doublet, the suboxide peaks are located at 16.93 and 18.63 eV, and they are associated with HfO_2−x_. The atomic concentration of hafnium in HfO_2_ thin film deposited at a low temperature by thermal ALD was the lowest at 23.7%, because many defects, such as carbon, nitrogen and hydroxyl groups (−OH), were located in the HfO_2_ thin films. Conversely, the atomic concentration of hafnium in HfO_2_ deposited at 80 °C by PEALD was high, at 30.0%, due to the low level of contaminants, similar to the sample deposited at 250 °C by thermal ALD. Figure 3c,d shows the XPS results for O 1 s after thermal ALD and PEALD. O 1 s peaks are deconvoluted into two components, a signal associated with HfO_2_ at 530.03 eV and an additional peak associated with carbon and oxygen at 531.68 eV [33]. The C–O peaks represent impurity carbon defects combined with oxygen, which can reduce the performance and efficiency of electronic devices [34,35]. According to these results, as the deposition temperature increased from 80 to 250 °C in both the thermal ALD and PEALD processes, the ratio of the C–O peaks showed a tendency to decrease. Furthermore, in the PEALD process, the ratio of C–O peaks was reduced compared with thermal ALD at all temperatures. In particular, at 80 °C in PEALD, C–O peaks decreased more than at 250 °C in thermal ALD. The atomic concentration of oxygen in HfO_2_ was similar, except for the thin film deposited at a low temperature by thermal ALD. As mentioned previously for elemental Hf, the presence of many contaminants can lower the atomic concentration of oxygen in thin films.

Figure 3e shows surface component percentages of O, Hf, C and N in the HfO_2_ thin films. Carbon and nitrogen inside the film act as defects, causing a decrease of density or degradation of properties. At a low temperature, HfO_2_ deposited through thermal ALD had high ratios of carbon (13.8%) and nitrogen (7.0%) because of incomplete source decomposition. Conversely, in the case of thin films deposited through PEALD, both carbon (3.5%) and nitrogen (2.8%) ratios were low, even at low temperatures. This suggests that, in PEALD, because the precursor was decomposed more by O_2_ plasma than by O_3_, the number of inner defects was lower than in thermal ALD at a low temperature. 

**Figure 4:**
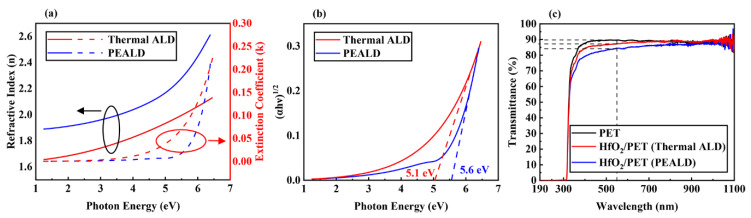
(**a**) Refractive index (n), extinction coefficient (k) and (**b**) optical bandgap values extracted using the Tauc method for HfO_2_ films (50 nm) formed at 80 °C, using thermal ALD and PEALD, as measured by ellipsometry. (**c**) Transmittance of HfO_2_ thin films (50 nm) deposited at 80 °C by thermal ALD and PEALD on PET substrate.

Figure 4a shows refractive index (n) and extinction coefficient (k) as a function of the photon energy (eV) of HfO_2_ films (50 nm) deposited at 80 °C. 

Using ellipsometry, values of n and k were calculated from the real and imaginary parts of the complex dielectric function (ε = ε_1_ + iε_2_), respectively [36]. The n values of the HfO_2_ are associated with the density of HfO_2_ thin films [37,38]. Since the HfO_2_ film deposited by PEALD had less carbon content and a lower O/Hf ratio than that obtained from using thermal ALD, it is expected that the HfO_2_ film deposited by PEALD has a higher density than that deposited by using thermal ALD. Therefore, the n value of the HfO_2_ deposited with PEALD was higher than that of HfO_2_ deposited with thermal ALD in all photon energy ranges. Figure 4b shows optical-band-gap values obtained from using the absorption coefficient (α = 4πk/λ) of HfO_2_ thin films (50 nm) deposited at 80 °C. The band gap of HfO_2_ thin film in the previous studies were typically between 5.6 and 5.7 eV [39,40]. However, the band gap of HfO_2_ deposited by thermal ALD was lower at 5.1 eV. When HfO_2_ was deposited by PEALD, the optical band gap increased to 5.6 eV. If the optical band gap is small, HfO_2_ thin films cannot function properly as insulators. Optical band gap was plotted by using the Tauc method, as described in Equation (1) [41]:

(1) $${\left(\alpha h\nu \right)}^{1/2}=A\left(h\nu -E_{g}\right)$$

where *α*(= 4*πκ/λ*) is the absorption coefficient, *h* is Planck’s constant, *ν* is photon frequency, *A* is a proportionality constant and *E_g_* is the optical band gap.

Figure 4c shows the transmittance of HfO_2_ (50 nm) deposited at 80 °C on PET substrate. The transmittance of HfO_2_ deposited by PEALD was high (>84%) in the visible region (89.7% for bare PET substrate, 87.2% after thermal ALD and 84.3% for PEALD at wavelength of 550 nm). The transmittance decreased slightly for PEALD compared to thermal ALD because the HfO_2_ film deposited by PEALD was denser [42]. When HfO_2_ was deposited at more than 80 °C on PET substrate, PET could not endure the heat, and deformation occurred. 

**Figure 5:**
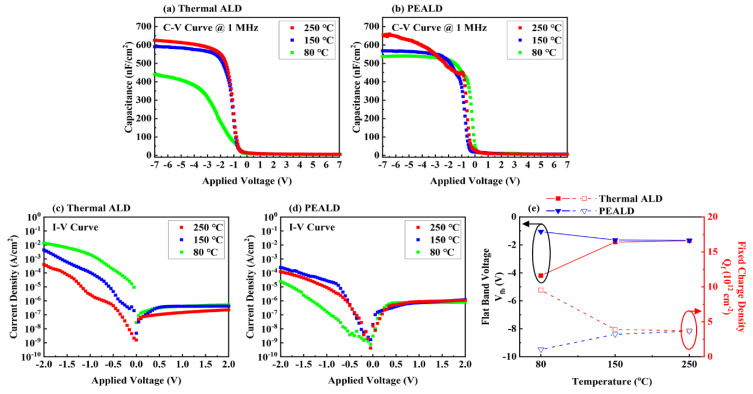
(**a**,**b**) C–V and (**c**,**d**) I–V curves of HfO_2_ (10 nm) MOS capacitors fabricated, using thermal ALD and PEALD. (**e**) Flat band voltage and fixed charge density of HfO_2_ (10 nm) MOS capacitor according to growth temperature (80–250 °C).

Figure 5a,b shows C–V curves of HfO_2_ deposited by thermal ALD and PEALD, respectively.

Capacitance of HfO_2_ deposited at 80 °C by PEALD increased from 444.9 to 540.1 nF/cm^2^, an approximately 21% increase. The dielectric constant (κ-value) of HfO_2_ deposited at 80 °C in PEALD (12.6) was higher than those of samples deposited by thermal ALD (8.7). Since the native oxide was not etched on the Si substrate, the κ-value was calculated by considering the native oxide thickness (~3 nm) [43,44]. Moreover, the κ-value of HfO_2_ thin film was calculated from the value of C_HfO2_, using the following formula, Equation (2):

(2) $$\frac{1}{C_{{\mathrm{HfO}}_{2}}}=\frac{1}{C_{\mathrm{ox}}}-\frac{1}{C_{{\mathrm{SiO}}_{2}}}$$

where C_HfO2_ and C_SiO2_ are the capacitance of HfO_2_ and SiO_2_, respectively. C_ox_ is the overall capacitance of the MOS capacitor.

There was no significant improvement at temperatures other than 80 °C. Figure 5c,d shows I–V curves of HfO_2_ deposited by thermal ALD and PEALD. Leakage currents at negative voltage in PEALD were reduced overall compared to those for thermal ALD. Significantly, at 80 °C, the leakage current decreased from 1.4 × 10^−2^ A/cm^2^ to 2.5 × 10^−5^ A/cm^2^ at −2 V, which was lower than that of HfO_2_ deposited at 250 °C by thermal ALD. Because HfO_2_ films deposited by PEALD at a low temperature were denser, contaminants in the thin films were reduced [45]. Because we used an NMOS capacitor with a p-type Si substrate, a depletion layer formed at the interface when the voltage was positive. For this sample, almost no current flowed, because the capacitor was in an inversion state.

Figure 5e shows the flat band voltage (V_fb_) and fixed charge density (Q_f_), extracted from the C–V curves in Figure 5a,b. V_fb_ of HfO_2_ deposited by PEALD at 80 °C was lower than those of sample formed by thermal ALD. Q_f_ of HfO_2_ deposited at 80 °C by PEALD decreased about 90%, from 9.5 × 10^12^ to 1.0 × 10^12^, the lowest value in all temperature ranges (80–250 °C). V_fb_ and Q_f_ showed almost identical values at all temperatures, except 80 °C. Q_f_ has a (+) charge and is distributed at the interface between Si and the HfO_2_ thin film, which puts the device into a (+) state and makes it to work at higher voltage. This suggests that, at low temperatures of around 80 °C, capacitors using HfO_2_ deposited by PEALD will have better electrical properties than those using HfO_2_ deposited by thermal ALD. However, the Q_f_ value of HfO_2_ deposited by PEALD tended to increase as the temperature increased. This means that, as the deposition temperature rose, the substrate was damaged by increased plasma ion energy during deposition [46,47].

## 4. Conclusions

In this study, HfO_2_ thin films deposited at a low temperature (80 °C), using PEALD with O_2_ plasma, showed improved properties compared to films deposited by using thermal ALD. The ALD window shifted from high temperatures (150–200 °C) to low temperatures (80–150 °C) when using PEALD, allowing stable deposition at a low temperature. HfO_2_ deposited by low-temperature PEALD showed a flat surface and higher density than films deposited by thermal ALD. Moreover, HfO_2_ deposited at 80 °C by PEALD showed a decreased presence of contaminants, such as carbon and nitrogen, compared to films deposited by thermal ALD. HfO_2_ thin films deposited by PEALD showed an increased refractive index, improved optical band gap (5.6 eV) and high transparency of ~84%. Denser and lower-contaminant HfO_2_ thin films deposited by PEALD contributed to capacitance improvement of about 21%, low leakage current of 2.5 × 10^−5^ A/cm^2^ and the lowest fixed charge density (1.0 × 10^12^). As a result, due to the higher decomposition power of O_2_ plasma, HfO_2_ thin films deposited at a low temperature by PEALD showed improved properties compared to those of films deposited by thermal ALD.
